# Preoperative position and protection of radial nerve by B-ultrasound combined with MIPPO for treatment of middle-inferior humerus fractures

**DOI:** 10.1186/s13018-022-03149-7

**Published:** 2022-05-12

**Authors:** Chaode Cen, Yongfei Cao, Yong Zhang, Chaoran Hu, Chunshan Luo

**Affiliations:** 1 Department of Orthopedics, The Guizhou Provincial Orthopedic Hospital, Guiyang, 550014 Guizhou China; 2 Department of Gynaecology and Obstetrics, Guiyang First People’s Hospital, Guiyang, 550002 Guizhou China

**Keywords:** Middle-inferior humerus fractures, Posterior approach MIPPO, Radial nerve, B-ultrasound

## Abstract

**Background and purpose:**

Open reduction and internal fixation through the posterior approach are standard methods for treating middle-inferior humerus fractures. Given the limited operative field and difficulty in locating the radial nerve, the minimally invasive percutaneous plate osteosynthesis (MIPPO) technique via the posterior approach to treat middle-inferior humerus fractures has rarely been reported. This study aims to evaluate the clinical effect of the preoperative study of the radial nerve position by B-ultrasound and its intraoperative protection combined with MIPPO in managing middle-inferior humerus fractures.

**Methods:**

The data were studied retrospectively involving 64 participants who had surgery for middle-inferior humerus fractures from the start of 2017 to the end of 2020. Participants were divided into two groups, those treated with the MIPPO technique, including newly developed dual procedures and preoperative position and protection of radial nerve by B-ultrasound (group A), and those treated with open reduction and internal plating fixation (group B).

**Results:**

All the cases were followed up for 12–34 months (an average of 25.6 ± 8.76 months), and there was no significant difference in the mean operative duration, surgical incision infection, range of motion (ROM) and MEPS (Mayo elbow performance score) for groups A and B. However, the occurrence of complications (radial nerve palsy, bone nonunion and flexible internal fixation or ruptures) in group B was significantly higher than the group A. A statistically significant difference was observed in the intraoperative blood loss, hospital stay and fracture nonunion time between the two groups. All the cases gained bone union within the MIPPO group.

**Conclusion:**

MIPPO via the posterior dual approach associated with preoperative position and protection of radial nerve by B-ultrasound does not increase radial nerve injury, however, it exhibits obvious advantages in the bone union, which is worthy of clinical application.

## Introduction

Humerus shaft fracture accounts for 1–1.5% of all fractures, and most of the fracture sites are located in the middle and lower segments [1]. Open reduction and internal fixation (OR/IF) are the most widely used surgical method for middle-inferior humerus fractures. However, the existing treatments of humerus fractures have limitations, such as invasiveness of the techniques and the development of numerous surgical scars that are difficult to repair, and the incidence of fracture nonunion tends to increase [2]. In addition, most of the impediments such as infections and radial nerve injury have been reported [3].

In managing long bone fractures, minimally invasive percutaneous plate osteosynthesis (MIPPO) offers an appealing alternative to existing surgical procedures such as open reduction with plate osteosynthesis or intramedullary nailing. For enhanced fracture healing, subtle indirect reduction procedures and percutaneous submuscular implantation of pre-contoured plates with locking screw technology for bridging plate fixation may protect the fracture hematoma and residual osseous blood supply [4, 5]. Nevertheless, this procedure also shows some drawbacks, such as the complexity of the approach, a small fixing area, and the danger of radial nerve damage. The procedure differs significantly depending on the location of fractures. For humeral proximal and diaphysial fractures, anterior and anterolateral methods are employed, while for middle-inferior humerus fractures, a posterior technique is employed [6].

To surgically treat middle-inferior humerus fractures, the most critical concern is how to expose the radial nerve and protect it. Previously, the radial nerve was fully exposed and prevented from being entrapped by adopting an enlarged incision. Nevertheless, the soft tissue blood supply at the fracture ends can be severely affected by severe surgical trauma and wide dissection of soft tissue, increasing the risk of nonunion and surgical incision site infection. Various cadaveric and clinical researches have addressed all sorts of elements of the radial nerve's anatomical proximity and potential damage [7–9].

With the increasing application of minimally invasive percutaneous fixation in managing distal humerus fractures, especially in recent years, the application of locking compression plates and adequate knowledge of anatomy have made this technique obtain satisfactory clinical results [10, 11].

Consequently, to overcome the shortcomings of existing conventional OR/IF and limitations of traditional MIPPO and solve the dual problems of the exposure of radial nerve and optimized surgical trauma, we developed a new MIPPO technique, performed via a posterolateral approach for the distal incision and a posterior approach for the proximal incision determined by B-ultrasound to identify the trajectory of the radial nerve. This retrospective study aimed to evaluate the clinical outcomes of two groups of patients, those treated with MIPPO using the newly proposed dual incision determined by B-ultrasound and those treated with traditional open reduction and internal plating fixation.

## Methods

### Patients’ selection

Patients with middle-inferior humerus fractures without radial nerve palsies were enrolled in the study group and were treated with either open reduction and plating osteosynthesis or the MIPPO approach. From the beginning of 2017 to the end of 2020, 72 patients who attended Guizhou Provincial Orthopedics Hospital to treat a middle-inferior humerus fracture and had the operation as mentioned above were retrospectively studied. The patient’s inclusion criteria were

1. A fracture located at least 3 cm proximal to the olecranon fossa;
2. A grade I or II open fracture or closed fracture;
3. No radial nerve injuries.

The exclusion criteria were

1. Combined with severe damage to other organs;
2. Fractures other than the middle-inferior humerus fractures;
3. Preoperative manifestations of radial nerve injury;
4. Juvenile patients whose epiphyses are not closed;
5. A grade III open fracture or closed fracture;
6. Patients combined with mental illness.

Sixty-four patients with isolated middle-inferior humerus fractures were matched for the inclusion criteria. The respondents were distributed into two groups in accordance with the random number table, patients treated with the MIPPO technique using the newly designed dual approaches (group A) and patients treated with open reduction and internal plating fixation (group B).

In group A, 32 patients (17 males and 15 females) ranged from 26 to 74 years, and the average age is 45.69 years. Twenty-one cases of falls or slips, nine cases of road accidents, and two patients with occupational injuries resulted in fractures. Five instances encountered composite damages in addition to the humeral fractures, including two cases of scapula and clavicle fractures, two patients with rib fractures, and one instance of elbow fracture.

The remaining 32 patients (14 males and 18 females) were allocated to group B, with an average age of 47.57 years (27–76 years). Twenty-two road accidents, six cases of falls or slips, and four industrial accidents were the causes of fracture. Seven instances were met with combined damage in addition to the humeral fractures. Scapula and clavicle fractures accounted for three instances, a rib fracture yielded two cases and an elbow fracture was two cases.

A 3.5 mm locked anatomical compression plate (XingRong Bolt®, Suzhou, Jiangsu, China) was utilized for all cases in group A and group B. The plates were pre-contoured prior to the surgery.

### Surgical technique

In cadaver experiments, Jiamton et al. [12] established a procedure that used a twofold incision while performing an open reduction. We applied the principle of this method to achieve the minimally invasive approach of MIPPO technique.

**Fig. 1:**
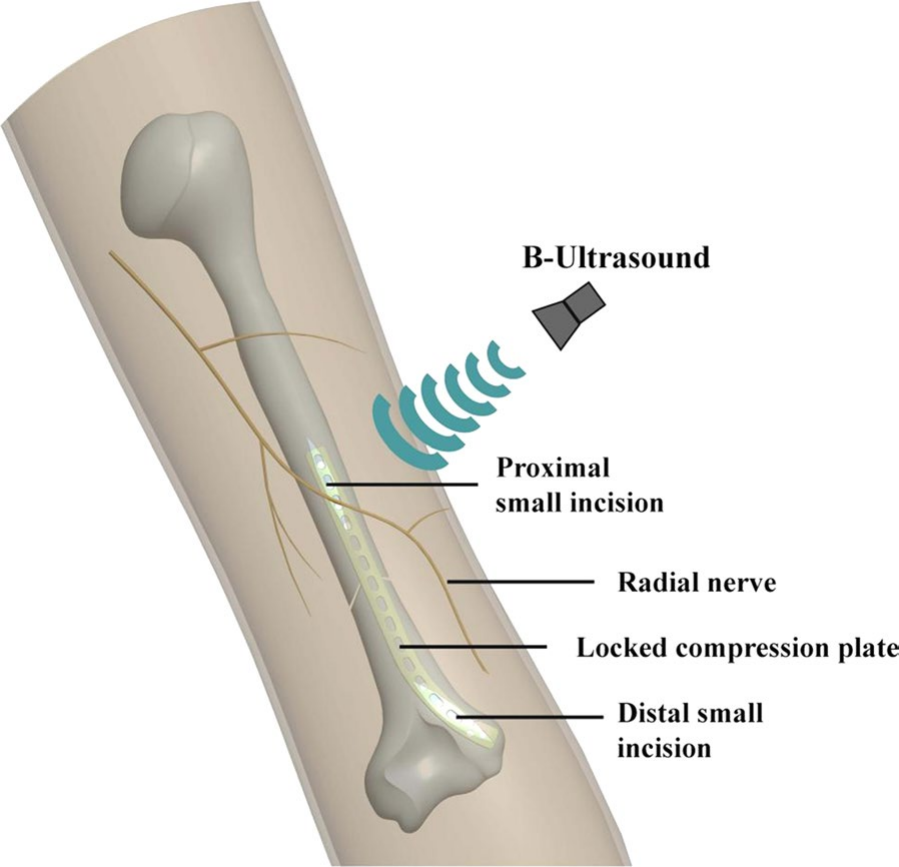
The schematic diagram of preoperative position and protection of radial nerve by B-ultrasound combined with MIPPO for treating middle-inferior humerus fractures

First, when brachial plexus block of the affected limb is effective, the treatment was performed with the body prone and the arm lying on a table at a 90-degree angle to the shoulder, allowing the elbow to be bent beyond 90 degrees and fully extended. Prior to the operation, the radial nerve was located in advance via B-ultrasound by an trained anesthesiologist with musculoskeletal ultrasound experience to facilitate the selection of proximal incision (Fig. 1).

Second, approximately 10 cm distal to the posterolateral point of the acromion, a 4 cm proximal incision was made in the posterior portion of the arm. The dissection then proceeded in line with the skin incision to visualize the deltoid's posterior boundary and the triceps' lateral head. The radial nerve and associated deep brachial artery were exposed by creating a gap between the long and lateral heads of the triceps. A vascular sling was used to separate and preserve the radial nerve properly. The radial nerve was methodically freed from the surrounding tissues to establish the proximal section of the plate tunnel.

Third, a 4 cm distal incision is made immediately proximal to the olecranon tip over the posterolateral portion of the arm. At the distal incision, the medial head of the triceps is longitudinally sliced and dragged inward to reveal the distal humerus. The forearm’s posterior branch of the lateral cutaneous nerve must be safeguarded throughout this procedure. The fracture was temporarily fixed and reduced with Kirschner wires after traction and rotary reduction, avoiding the plate placement area as far as possible. A sub-triceps extra-periosteal tunnel was formed from the distal to the proximal incision.

Fourth, from the distal incision to the proximal incision, a 10-hole 3.5 mm extra-articular distal humeral locked compression anatomical plate ((XingRong Bolt®, Suzhou, Jiangsu, China) was closely attached to the humerus. The radial nerve was held off the humerus using a sling to prevent entrapped when the plate was placed. Elbow flexion and forearm pronation were supposed to ease radial nerve strain. The plate was placed on the lateral column between the olecranon fossa and the lateral border of the bone at the distal incision and secured with one screw. It was moved around this screw via the proximal incision until it was centrally placed on the posterior part of the humerus, at that point, a proximal screw was placed under direct eyesight. Finally, residual screws for proximal and distal fixation were placed. After the fracture reduction was successfully performed, the elbow joint was passively moved without entrapment or abnormal sound, and the fracture was stably fixed. The incision was completely stanched, rinsed and closed, and the drainage tube was placed. A muscle flap (partial lateral head of triceps brachii) may be put here between the plate and the radial nerve to minimize radial nerve discomfort, which also can protect the radial nerve from wrapping and stimulating by callus, making it difficult to detect the radial nerve when the plate was removed.

### Postoperative evaluation

The clinical outcome was assessed by the mean operative duration, intraoperative blood loss, hospital stay, surgical incision site infection, bone healing time, radial nerve injury, bone nonunion, flexible internal fixation and Mayo elbow performance score, and the range of elbow joint. The radiographic data were used to assess the success of the union, the length of the union, and the alignment of the bones. Follow-up examinations were done at 3 months, 6 months, 9 months, 1 year, and 2 years following surgery.

### Statistical analysis

SPSS software version 13.0 was used for statistical analysis. The quantitative data were expressed as the mean ± standard deviation. *T* test was used for intergroup comparison. The counting data were expressed as number and percentage (%), and the Pearson’s chi-square test analyzed the intergroup comparison. A *P* value < 0.05 was considered statistically significant.

## Results

The average operation time in group A was 75 ± 20 min, and the mean operating time in group B was 80 ± 10 min, the difference between the two groups was not considered significant. The average intraoperative blood loss in group A was 140 ± 20 mL, while that in group B was 190 ± 15 mL, the alteration between the two groups showed significant difference (*P* = 0.0001 < 0.05). There was no surgical incision site infection in the two groups. There was a statistically considerable difference in average hospital stay, for group A it was 2.42 ± 1.28 days (ranging 1–6 days), while for group B, it was 3.36 ± 1.29 days (ranging 2–9 days). All the cases were followed up for 12–34 months (a mean of 25.6 ± 8.76 months), and the average fracture union time was 12.29 ± 3.22 weeks (ranging 10–24) in group A and 15.04 ± 3.35 weeks (ranging 10–24) in group B, all the cases in group A healed well. The average fracture union time in group B was longer than in group A, and there was a statistically significant difference between the two groups (*t* = 3.3479, *P* = 0.0014) (Table 1).

**Table 1:** Comparison of perioperative period clinical indexes between the two groups ($\overline{X} \pm S$)

Group	Operative duration (min)	Intraoperative blood loss (mL)	Hospital stay (days)	Fracture union time (weeks)
A	75 ± 20	140 ± 20	2.42 ± 1.28	12.29 ± 3.22
B	80 ± 10	190 ± 15	3.36 ± 1.29	15.04 ± 3.35
*P*	0.2106	0.0001	0.0048	0.0014
*t*	1.2649	11.3137	2.9260	3.3479

Radial nerve palsy occurred in one case in group A. There were three cases of radial nerve palsy, five cases of bone nonunion and one case of internal fixation loosening in group B. The occurrence of complications (radial nerve palsy, bone nonunion and flexible internal fixation or ruptures) in group B was higher than that in group A and showed a statistically significant difference between the two groups (*χ*^2^ = 7.5852, *P* = 0.0059; Table 2).

**Table 2:** Comparison of the occurrence of postoperative complications between the two groups

Group	Radial nerve palsy	Surgical incision infection	Bone nonunion	Flexible internal fixation or ruptures	Complications incidence (%)
A (*n* = 32)	1	0	0	0	3.13
B (*n* = 32)	3	0	5	1	28.13
*χ*^2^	0.2667				7.5852
*P*	0.6056				0.0059

No significant difference was observed in ROM and MEPS between the two groups. The mean ROM of flexion was 133.45 ± 9.35° (ranging 110°–145°) in group A and 129.50 ± 10.68° (ranging 112°–138°) in group B (*t* = 1.5742, *P* = 0.1205), the mean ROM of extension was − (7.55 ± 2.43)° (ranging from − 5° to − 10°) in group A and− (6.96 ± 2.29)° (ranging from − 4° to − 9°) in group B (*t* = 0.9996, *P* = 0.3214). The mean MEPS was 98.32 ± 2.57 points (ranging 90–100) in group A and 99.37 ± 1.78 points (ranging 95–100) (*t* = 1.9000, *P* = 0.0621) in group B. Insignificant irregularities were found after surgery in each group (Table 3).

**Table 3:** Comparison of the functional recovery of elbow joint after operation between the two groups

Group	ROM (degrees)		MEPS (points)
	Flexion	Extension	
A (*n* = 32)	133.45 ± 9.35	− (7.55 ± 2.43)	98.32 ± 2.57
B (*n* = 32)	129.50 ± 10.68	− (6.96 ± 2.29)	99.37 ± 1.78
*P*	0.1205	0.3214	0.0621
*t*	1.5742	0.9996	1.9000

**Fig. 2:**
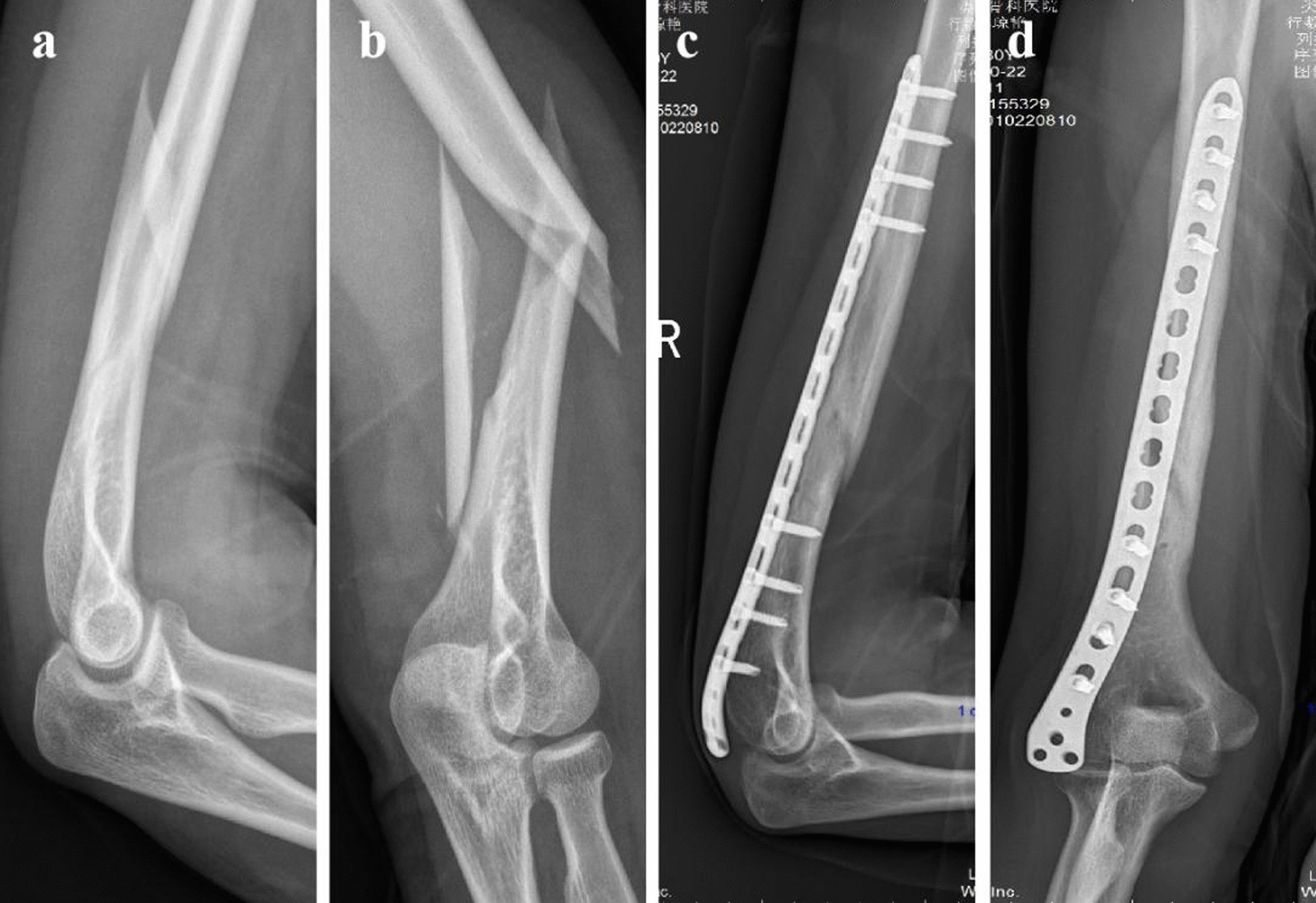
The MIPPO used in Case 1: a 31-year-old female, indirect violence in a fall leads to the right middle-inferior humerus fractures. **a** Preoperative lateral X-ray radiograph of the middle-inferior humerus fractures. **b** Preoperative anteroposterior X-ray radiograph of the middle-inferior humerus fractures. **c** The lateral X-ray radiograph of full-length humerus 8 months after MIPPO. **d** The anteroposterior X-ray radiograph of the full-length humerus 8 months after MIPPO

**Fig. 3:**
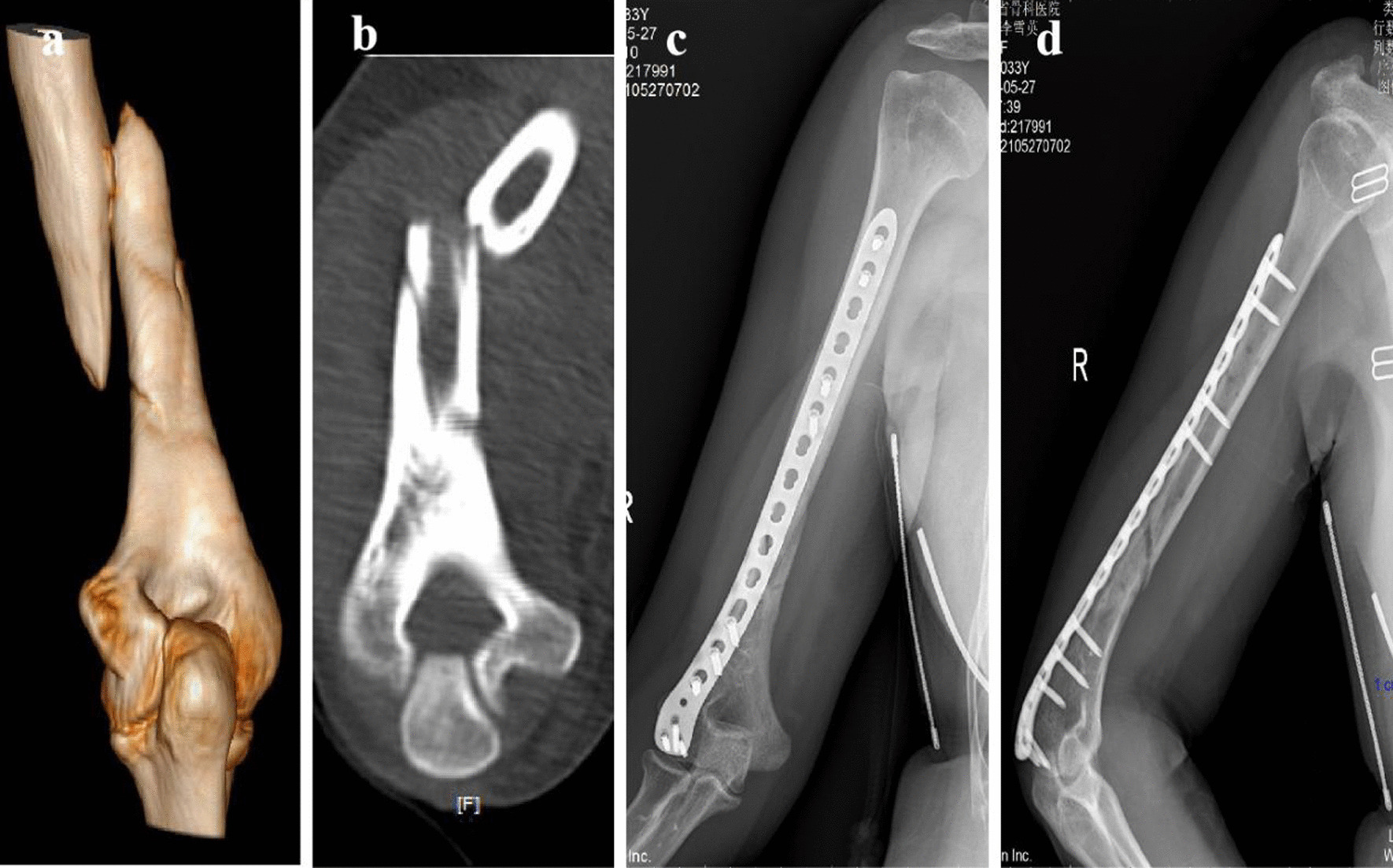
The MIPPO used in Case 2: a 34-year-old male, indirect violence in a fall leads to the right middle-inferior humerus fractures. **a** Preoperative 3-D CT imaging of the middle-inferior humerus fractures. **b** Preoperative CT coronal imaging of the middle-inferior humerus fractures. **c** The anteroposterior X-ray radiograph of the full-length humerus 2 months after MIPPO. **d** The lateral X-ray radiograph of the full-length humerus 2 months after MIPPO

**Fig. 4:**
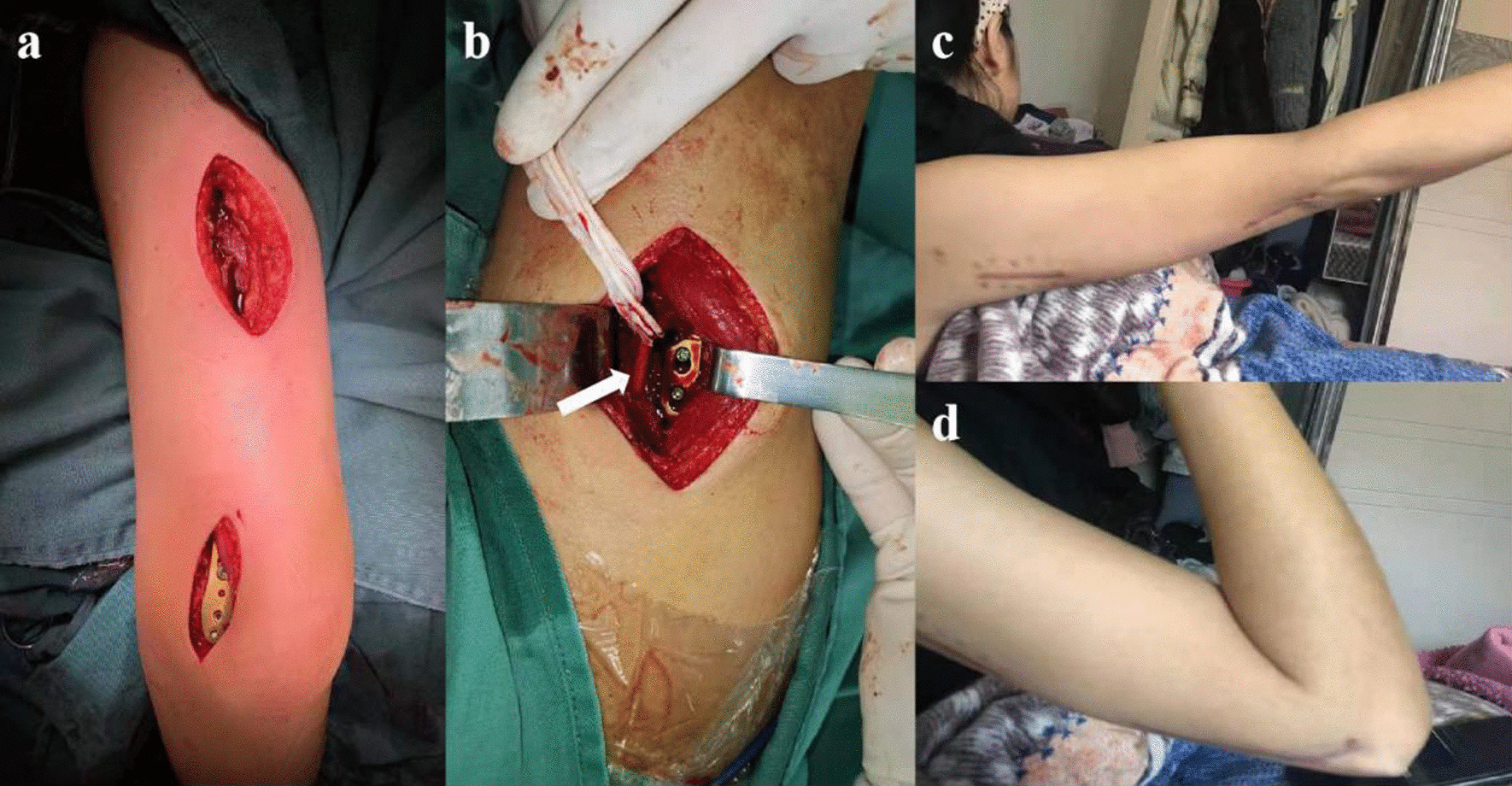
The selection and design of minimally invasive incision and postoperative recovery of MIPPO. **a** The appearance of an operative incision. **b** The radial nerve is exposed in the proximal incision (the arrow indicates the radial nerve). **c** The right elbow extension function and flexion function (**d**) are satisfactory

Typical cases present preoperative and postoperative follow-up imaging data of MIPPO technique for the treatment of middle-inferior humerus fractures, preoperative data suggest these cases meet inclusion criteria. The assistance of B-ultrasound established the upper arm dual invasive incision via posterior and posterolateral approach, the radial nerve is carefully exposed and protected in the proximal incision, good reduction and fixation were obtained, callus growth is evident at the fracture end during follow-up, the patient achieved satisfactory flexion and extension function of the elbow joint (Figs. 2, 3 and 4).

## Discussion

The middle-inferior humerus fractures are often caused by direct or indirect rotational force. The fracture pattern differs between young male patients with high-energy trauma and older female patients with an osteoporotic bone structure in low-energy trauma, indicating a bimodal distribution [13]. The incidence of fractures has increased with the increase in traffic accidents and fall events in recent years, seriously affecting the eminence of life and the health of patients.

The management of humeral diaphysial fracture is debatable and remains a challenge in traumatology. As the excellent compensatory function of the shoulder joint, conservative treatment was considered one of the utmost generally recognized methods of managing humerus shaft fracture. After long-term follow-up, shoulder stiffness and increased occurrence of late or non or mal-union caused by prolonged immobilization and instability caused significant pain to patients, conservative treatment is suitable for the elderly and patients with contraindications to surgery [14]. Antegrade or retrograde humeral nails were successfully used to treat the humeral SF (shaft fracture), which correlated with increased overdue therapy, nonunion, injury of the rotator cuff muscles, and even failure [15, 16]. Classical clinical trials of OR/IF of long bones have exacerbated this situation due to iatrogenic damage to the periosteum blood supply and wide-ranging soft tissue dissection [17].

Numerous studies have reported this approach, encompassing the posterior MIPPO method for repairing fractures in the distal third humerus shaft and the anterior MIPPO method to manage humeral diaphyseal fracture and obtain biological fixation while minimizing the possible hurdles correlated with an open reduction [18–20]. Nonetheless, the radial nerve injury incidence marks it as a potentially hazardous surgical technique [21, 22].

At present, there are many literature works on the anatomical location of the radial nerve. For instance, Apivatthakakul et al. [10] reported a cadaveric study on MIPPO of the humerus through an anterior approach, the average distance from the radial nerve to the part of the plate that is nearest the nerve is 3.2 mm when the forearm is supinated. In another cadaveric study, Jiamton [12] determined the feasibility of applying MIPPO of the humerus via the posterior approach, the radial nerve could be elevated from the radial sulcus to a minimum height of 18 mm, which is far greater than the thickness of the steel plate. The radial nerve crossed the medial and lateral borders of the posterior surface of the humerus at 80.1–132 mm (average 104.7 mm) and 116.6–175.5 mm (average 142.7 mm) of its total length, respectively. Michael Hackl et al. [7] confirmed through a cadaver study that the absolute “safe zones” of the radial nerve in relation to the olecranon fossa are 10.5 and 7.5 cm at the medial and lateral edge of the humerus, respectively, the nerve is located at a minimum of 9 cm proximal to the olecranon fossa at the midshaft level. These anatomical data can be used as a reference to lower the occurrence of iatrogenic radial nerve injury. However, it is relatively reliable because these figures vary by different races and heights, and the radial nerve anatomy might be changed in trauma.

B-ultrasound is a non-invasive examination method, which has been applied to the diagnosis of many diseases in orthopedics because of its advantages such as no radiation, easy operation and low price [23]. Since the different acoustic impedances of the soft tissue structures to produce discrepant echoes, the identification of the radial nerve is obvious. B-ultrasound is an operator-dependent method, preoperative localization should be performed gently and requires highly skilled ultrasonographer to quickly locate the radial nerve. Fortunately, we have a group of anesthesiologists professionally trained in musculoskeletal ultrasound. Preoperative examinations after anesthesia did not aggravate the patient’s pain and discomfort. Preoperative B-ultrasound can locate the radial nerve crossing the radial nerve groove of the humerus, and the small proximal incision is made precisely to expose the radial nerve based on the principle. Preoperative precise positioning and intraoperative gentle pulling can reduce iatrogenic injury of the radial nerve, improving the efficiency of MIPPO. Given the extreme closeness of the radial nerve, MIPPO utilizing the posterior route to the humerus appears to be potentially dangerous. However, Balam et al. [4] proved the safety of this operation in 37 humeral shaft fracture patients. Our findings further support the safety of the posterior MIPPO technique.

Given the limited area provided by the coronoid and anconal fossas, as well as the comparatively poor bone quality in the metaphyseal area, adequate bone holding with at least three screws can be difficult to accomplish in middle-inferior humerus fractures that stretch more distally and have a very short distal fragment [24]. The implant should be adjusted, or an alternative approach should be used to avoid these issues. When treating middle-inferior humerus fractures, the posterior technique is usually utilized. This technique offers various advantages, including a flat surface suited for plate attachment, the possibility to position the plate further distally on the lateral column for increased stability, and the preservation of triceps morphology and function to promote quick postoperative therapy [25].

This study compared and analyzed the effects of open reduction and MIPPO in managing middle-inferior humerus fractures. The differences of mean operative duration, surgical incision infection, range of motion (ROM) and MEPS (Mayo elbow performance score) for groups A and B were insignificant. However, the occurrence of complications (radial nerve palsy, bone nonunion and flexible internal fixation or ruptures) in group B was significantly higher than in group A. There was a statistically significant difference in the intraoperative blood loss, hospital stay and fracture union time between the two groups.

Compared to MIPPO, traditional open reduction and internal fixation severely disrupt the blood supply to the fracture due to extensive soft tissue and periosteum dissection and even interference with the main nourishing artery of the humeral shaft, increasing the risk of fracture nonunion. Continued fretting of the fracture ends with nonunion and delayed union results in loosening and failure of the fixation. In this study, one case of radial nerve palsy occurred in group A, which may be related to the insufficient exposure and release of the radial nerve in a proximal incision. Fortunately, this radial nerve palsy was recovered 2 months after surgery. Open reduction internal fixation is associated with a higher risk of radial nerve palsy, which may be attributed to excessive traction, rough manipulation and lax surgical thinking caused by broad surgical space. However, there was no statistical difference in the incidence of radial nerve palsy between the two groups. The MIPPO technique did not increase the risk of radial nerve palsy due to a small surgical field but reduced surgical trauma and the incidence of related complications, these were attributed to the precisely preoperative position and protection of radial nerve by B-ultrasound.

The radial nerve was placed on the internal fixation surface when the incision was closed, to avoid the irritating injury of the radial nerve or being wrapped by the callus, which would make it challenging to identify the radial nerve when the internal fixation was taken out. The lateral triceps cephalic muscle flap could be transferred and fixed between the steel plate and the radial nerve to protect the radial nerve [26]. Nevertheless, given the postoperative tissue adhesion and originally limited surgical space, we believe that there is still a certain risk of radial nerve injury during the removal of the internal fixator, which requires careful intraoperative dissection to avoid. Therefore, one of the limitations of this study is that this technique is not always suitable for patients who require the removal of the internal fixation after fracture healing. The patients nor we are willing to accept that existing excellent outcomes are undermined by unnecessary internal fixation removal. However, there have been exceptions to that rule, the removal of internal fixation is inevitable in the events of severe and unpredictable complications such as implant-related infection, refracture, and fracture nonunion. In such cases, careful dissection, delicate operation, and dilating the incision appropriately can often avoid iatrogenic damage to the radial nerve. Moreover, this technique uses a bone plate as a template for indirect reduction and fixation, requiring some technical experience and a long learning curve. In addition, to verify its long-term clinical efficacy and increase its popularity, we also have to introduce this method to young orthopedic specialists at various trauma centers.

## Conclusions

MIPPO is an effective and valuable approach used for shaft fracture surgery. The main beliefs of the MIPPO method can be practicalized securely to manage the middle-inferior humerus fractures through the posterior percutaneous approach. MIPPO via the posterior and posterolateraldual approach associated with preoperative position and protection of radial nerve by B-ultrasound does not increase radial nerve injury, but exhibits obvious advantages in the bone union, which is worthy of clinical application. We believe that the current results may be of great importance and interest to the readers and clinicians.

## Data Availability

The datasets used and/or analyzed during the current study are available from the corresponding author on reasonable request.

## Abbreviations

MIPPO: Minimally invasive percutaneous plate osteosynthesis
ROM: Range of motion
MEPS: Mayo elbow performance score
OR/IF: Open reduction and internal fixation
SF: Shaft fracture
